# Supplementary material for: A Long-Term Study on the Content of Polycyclic Aromatic Hydrocarbons in Rubber from End-of-Life Tires of Passenger Cars and Trucks
Source: Materials (Basel). 2022 Oct 10;15(19):7017. doi: 10.3390/ma15197017 (PMC9571790; doi:10.3390/ma15197017)
Supplement: Supplementary file 1 [file materials-15-07017-s001.zip › materials-1870906-supplementary.pdf]

Supplementary Materials

A Long-Term Study on the Content of Polycyclic Aromatic Hydrocarbons in Rubber from End-of-life Tires of Passenger Cars and Trucks

Stefan Hoyer [1,*], Kirsten Lippert [2], Albrecht Seidel [2] and Lothar Kroll [1]

[1] Technische Universität Chemnitz, Professur Strukturleichtbau und Kunststoffverarbeitung, Reichenhainer Straße 31/33, 09126 Chemnitz, Germany
[2] Biochemical Institute for Environmental Carcinogens Prof. Dr. Gernot Grimmer-Foundation, 22927 Grosshansdorf, Germany
* Correspondence: stefan.hoyer@mb.tu-chemnitz.de (S.H.); Tel.: +49 371 531-37814

Table of content

Table S1. Limit of quantification (LOQ, µg/kg) and limit of detection (LOD, µg/kg) values using a sample amount of 10.00 g, and coefficients of determination (R^2) of the calibrations. page 2

Table S2. Overview of all individual measurement results for the PAH content of passenger car tires, wohle tires (PW) in mg/kg (part 1) page 3–4

Table S3. Overview of all individual measurement results for the PAH content of truck tires, whole tires (TW) in mg/kg page 5–6

Table S4. Overview of all individual measurement results for the PAH content of truck tires, tread (TT) in mg/kg page 7

Table S1. Limit of quantification (LOQ, µg/kg) and limit of detection (LOD, µg/kg) values using a sample amount of 10.00 g, and coefficients of determination (R^2) of the calibrations.

PAH	LOQ	LOD	R^2
Naphthalene	< 1.068	< 0.356	0.999
Acenaphthylene	< 0.279	< 0.093	0.999
Acenaphthene	< 0.137	< 0.046	0.999
Fluorene	< 0.029	< 0.010	0.999
Phenanthrene	< 0.016	< 0.005	0.999
Anthracene	< 0.005	< 0.002	0.995
Fluoranthene	< 0.009	< 0.003	0.999
Pyrene	< 0.027	< 0.009	0.999
Benzo[*b*]naphtho[2,1-*d*]thiophene	< 0.016	< 0.005	0.994
Benzo[*ghi*]fluoranthene	< 0.033	< 0.011	0.998
Benzo[*c*]phenanthrene	< 0.015	< 0.005	0.999
Benzo[*a*]anthracene	< 0.029	< 0.010	0.999
Cyclopenta[*cd*]pyrene	< 0.033	< 0.011	0.975
Triphenylene	< 0.016	< 0.005	0.999
Chrysene	< 0.016	< 0.005	0.999
Benzo[*b*]fluoranthene	< 0.006	< 0.002	0.999
Benzo[*k*]fluoranthene	< 0.006	< 0.002	0.997
Benzo[*j*]fluoranthene	< 0.006	< 0.002	0.997
Benzo[*e*]pyrene	< 0.024	< 0.008	0.996
Benzo[*a*]pyrene	< 0.042	< 0.014	0.999
Perylene	< 0.024	< 0.008	0.990
Indeno[1,2,3-cd]pyrene	< 0.027	< 0.009	0.990
Dibenzo[*a,h*]anthracene	< 0.011	< 0.004	0.995
Benzo[*ghi*]perylene	< 0.019	< 0.006	0.999
Anthanthrene	< 0.019	< 0.006	0.960
Coronene	< 0.019	< 0.006	0.999

Table S2. Overview of all individual measurement results for the PAH content of passenger car tires, wohle tires (PW) in mg/kg (part 1)

**Date**	**07.17**		**08.17**				**09.17**			**10.17**		**11.17**		**12.17**		**01.18**		**02.18**		**03.18**	
**Sample number**	1	2	3	4	5	6	7	8	9	10	11/12	13 (A)	13 (B)	14 (A)	14 (B)	15 (A)	15 (B)	16 (A)	16 (B)	17 (A)	17 (B)
**PAH species**	*mg/kg*	*mg/kg*	*mg/kg*	*mg/kg*	*mg/kg*	*mg/kg*	*mg/kg*	*mg/kg*	*mg/kg*	*mg/kg*	*mg/kg*	*mg/kg*	*mg/kg*	*mg/kg*	*mg/kg*	*mg/kg*	*mg/kg*	*mg/kg*	*mg/kg*	*mg/kg*	*mg/kg*
Naphthalene	1.578	1.531	1.375	1.652	1.707	1.477	2.181	2.494	1.676	1.527	1.500	1.471	1.401	2.080	2.540	3.388	3.200	1.448	1.531	1.388	1.342
Acenaphthylene	0.937	0.994	1.017	1.022	0.990	1.084	1.050	1.190	0.857	1.042	1.245	1.190	1.077	1.121	1.101	1.107	1.002	1.349	1.335	1.248	1.291
Acenaphthene	0.082	0.162	0.868	0.158	0.085	0.139	0.074	0.071	0.072	0.125	0.546	0.104	0.097	0.077	0.070	0.081	0.079	0.140	0.130	0.132	0.124
Fluorene	0.247	0.320	0.794	0.269	0.318	0.252	0.256	0.252	0.243	0.287	0.457	0.245	0.239	0.248	0.260	0.249	0.258	0.384	0.348	0.309	0.290
Phenanthrene	3.855	4.030	6.289	3.849	4.295	3.750	3.691	3.723	3.882	4.004	4.851	3.851	3.734	4.032	3.954	3.700	3.729	4.901	4.875	4.312	4.394
Anthracene	0.251	0.254	0.339	0.279	0.368	0.322	0.265	0.248	0.295	0.323	0.302	0.230	0.246	0.260	0.283	0.273	0.270	0.390	0.365	0.321	0.321
Fluoranthene	7.714	7.876	9.042	7.702	8.322	7.522	7.656	8.249	8.280	8.148	8.744	7.946	7.806	8.450	8.204	7.753	7.824	9.694	9.706	8.503	8.746
Pyrene	30.162	30.336	30.885	29.842	29.413	29.255	29.845	31.474	30.446	29.414	32.429	31.504	31.091	32.206	31.356	30.357	30.869	33.606	34.102	31.349	32.370
Benzo[*b*]naphtho[2,1-*d*]thiophene	0.983	1.089	1.050	1.013	0.923	1.113	1.068	0.980	1.470	1.148	0.869	0.907	0.888	1.450	1.525	0.984	0.954	1.400	1.283	1.168	1.141
Benzo[*ghi*]fluoranthene	3.557	3.555	3.593	3.509	3.461	3.521	3.347	3.557	3.598	3.345	3.474	3.333	3.357	3.767	3.762	3.461	3.556	3.651	3.443	3.350	3.411
Benzo[*c*]phenanthrene	0.182	0.179	0.205	0.196	0.220	0.163	0.204	0.215	0.235	0.210	0.200	0.177	0.180	0.222	0.238	0.210	0.209	0.291	0.258	0.246	0.225
Benzo[*a*]anthracene	0.409	0.491	0.415	0.471	0.707	0.487	0.456	0.467	0.619	0.566	0.433	0.345	0.334	0.444	0.505	0.475	0.457	0.702	0.681	0.545	0.605
Cyclopenta[*cd*]pyrene	4.564	4.258	3.946	4.403	4.738	5.425	4.293	4.853	4.400	4.124	4.723	4.525	4.439	5.136	5.105	4.910	4.979	5.311	4.677	5.159	5.066
Triphenylene	1.047	1.200	1.069	1.071	0.905	1.142	1.144	1.063	1.536	1.038	0.829	0.916	0.851	1.399	1.521	0.961	0.905	1.381	1.250	1.133	1.177
Chrysene	0.928	1.093	0.963	1.010	1.061	1.055	1.032	1.117	1.374	1.103	0.860	0.807	0.766	1.149	1.219	0.971	0.928	1.343	1.334	1.105	1.185
Benzo[*b*]fluoranthene	0.870	0.913	0.817	0.884	1.043	0.972	0.868	0.956	1.142	0.952	0.778	0.777	0.745	1.087	1.114	0.886	0.856	1.213	1.159	0.957	1.054
Benzo[*k*]fluoranthene	0.271	0.282	0.233	0.280	0.344	0.271	0.232	0.303	0.370	0.332	0.242	0.239	0.245	0.328	0.389	0.267	0.296	0.368	0.366	0.323	0.337
Benzo[*j*]fluoranthene	0.206	0.257	0.215	0.253	0.353	0.275	0.264	0.299	0.284	0.284	0.251	0.222	0.213	0.304	0.291	0.303	0.283	0.404	0.394	0.287	0.325
Benzo[*e*]pyrene	3.148	3.248	2.992	3.078	2.967	3.356	2.891	2.949	3.605	2.914	2.613	2.752	2.676	3.654	3.735	3.024	2.983	3.606	3.484	3.206	3.332
Benzo[*a*]pyrene	1.808	1.831	1.708	1.788	1.968	1.901	1.686	1.819	1.886	1.784	1.676	1.642	1.609	1.895	1.889	1.851	1.821	2.088	2.058	1.901	2.031
Perylene	0.432	0.433	0.381	0.407	0.480	0.515	0.399	0.405	0.517	0.419	0.357	0.360	0.349	0.490	0.533	0.439	0.430	0.526	0.495	0.464	0.496
Indeno[1,2,3-*cd*]pyrene	2.280	2.209	2.157	2.252	2.387	2.464	1.982	2.213	2.202	2.100	2.117	2.150	2.097	2.440	2.403	2.372	2.327	2.691	2.653	2.452	2.623
Dibenzo[*a,h*]anthracene	0.066	0.069	0.047	0.060	0.078	0.095	0.065	0.074	0.061	0.043	0.061	0.054	0.042	0.048	0.071	0.050	0.055	0.119	0.125	0.091	0.086
Benzo[*ghi*]perylene	14.072	13.681	13.632	13.397	12.866	13.461	12.749	13.497	13.497	12.876	12.636	12.980	12.741	14.119	13.678	12.850	13.216	14.212	14.312	13.698	14.045
Anthanthrene	4.407	3.980	3.812	4.123	4.420	4.765	3.805	4.152	4.175	4.020	3.972	4.050	4.065	4.880	4.618	4.384	4.680	4.521	4.065	4.249	4.298
Coronene	12.552	10.730	11.539	11.788	10.727	12.187	10.620	11.349	11.257	11.100	11.262	11.766	12.004	12.818	12.388	12.365	12.341	11.650	11.088	11.233	11.401

Table S2. Overview of all individual measurement results for the PAH content of passenger car tires, wohle tires (PW) in mg/kg (part 2)

Date	04.18		05.18		06.18		07.18		08.18	09.18	10.18	11.18	12.18	01.19	02.19	03.19	04.19	05.19	06.19	07.19
**Sample number**	18 (A)	18 (B)	19 (A)	19 (B)	20 (A)	20 (B)	21 (A)	21 (B)	22	23	24	25	26	27	28	29	30	31	32	33
**PAH species**	*mg/kg*	*mg/kg*	*mg/kg*	*mg/kg*	*mg/kg*	*mg/kg*	*mg/kg*	*mg/kg*	*mg/kg*	*mg/kg*	*mg/kg*	*mg/kg*	*mg/kg*	*mg/kg*	*mg/kg*	*mg/kg*	*mg/kg*	*mg/kg*	*mg/kg*	*mg/kg*
Naphthalene	1.213	1.238	1.391	1.194	1.562	1.505	1.568	1.504	1.153	1.261	1.211	1.883	2.129	1.804	1.134	1.156	1.260	1.067	1.097	1.473
Acenaphthylene	1.171	1.199	1.334	1.135	1.212	1.119	1.185	1.263	1.037	1.219	1.257	1.301	1.281	1.193	1.100	1.103	1.182	1.076	1.067	1.274
Acenaphthene	0.100	0.106	0.130	0.117	0.100	0.107	0.124	0.135	0.053	0.065	0.081	0.096	0.082	0.099	0.089	0.070	0.086	0.064	0.063	0.064
Fluorene	0.269	0.299	0.337	0.311	0.282	0.268	0.336	0.331	0.234	0.235	0.196	0.455	0.401	0.400	0.197	0.176	0.231	0.168	0.235	0.172
Phenanthrene	3.965	3.984	4.415	4.137	4.049	3.865	4.118	4.304	3.792	3.981	3.948	5.685	5.235	5.003	4.077	3.463	4.444	3.443	4.134	3.932
Anthracene	0.296	0.273	0.325	0.310	0.296	0.292	0.323	0.325	0.236	0.240	0.215	0.536	0.432	0.409	0.328	0.221	0.361	0.240	0.353	0.357
Fluoranthene	8.249	8.323	8.902	8.579	8.770	8.389	8.732	8.937	8.482	7.964	7.751	9.936	9.080	8.677	7.486	6.770	8.089	6.848	7.484	7.488
Pyrene	31.434	31.903	32.663	31.529	32.894	31.687	32.087	33.005	31.662	30.552	29.978	33.512	32.828	31.454	24.020	23.792	25.299	23.443	24.605	25.394
Benzo[*b*]naphtho[2,1-*d*]thiophene	1.141	1.183	0.942	0.952	1.338	1.306	1.111	1.043	0.754	0.548	0.510	0.783	0.622	0.623	0.911	0.625	1.060	0.586	0.610	0.661
Benzo[*ghi*]fluoranthene	3.489	3.549	3.482	3.364	3.746	3.570	3.462	3.449	3.021	2.914	2.793	3.203	3.201	2.932	2.926	2.813	3.079	2.723	2.815	2.990
Benzo[*c*]phenanthrene	0.209	0.213	0.226	0.232	0.256	0.239	0.268	0.247	0.184	0.146	0.161	0.229	0.185	0.185	0.216	0.174	0.228	0.164	0.183	0.138
Benzo[*a*]anthracene	0.452	0.432	0.538	0.575	0.480	0.504	0.669	0.545	0.495	0.334	0.311	1.176	0.492	0.608	0.576	0.407	0.737	0.317	0.362	0.466
Cyclopenta[*cd*]pyrene	4.951	4.743	5.212	4.959	5.516	5.132	5.140	5.315	3.824	3.688	3.380	4.490	4.775	4.636	3.286	3.468	4.285	3.432	3.451	5.337
Triphenylene	1.112	1.192	0.893	0.920	1.152	1.128	1.139	1.014	0.901	0.563	0.646	1.027	0.806	0.810	0.985	0.733	1.111	0.744	0.590	0.735
Chrysene	0.976	0.997	0.932	0.986	1.032	1.022	1.200	1.003	0.927	0.600	0.635	1.493	0.904	0.948	1.083	0.803	1.316	0.743	0.686	0.840
Benzo[*b*]fluoranthene	0.910	0.935	0.938	0.988	0.978	0.967	1.010	0.909	0.879	0.661	0.629	1.441	0.832	0.926	0.931	0.704	1.059	0.662	0.648	0.816
Benzo[*k*]fluoranthene	0.276	0.276	0.309	0.337	0.310	0.284	0.346	0.284	0.254	0.198	0.134	0.459	0.221	0.244	0.311	0.232	0.361	0.196	0.201	0.285
Benzo[*j*]fluoranthene	0.270	0.243	0.323	0.312	0.284	0.298	0.314	0.308	0.274	0.181	0.206	0.564	0.268	0.303	0.340	0.231	0.366	0.201	0.201	0.271
Benzo[*e*]pyrene	3.202	3.262	2.994	3.052	3.307	3.273	3.251	2.981	2.684	2.256	2.241	3.301	2.631	2.619	2.556	2.248	2.760	2.148	2.032	2.358
Benzo[*a*]pyrene	1.849	1.838	1.859	1.937	1.927	1.873	1.993	1.917	1.719	1.521	1.458	2.503	1.785	1.811	1.706	1.523	1.895	1.458	1.450	1.835
Perylene	0.450	0.454	0.445	0.433	0.468	0.444	0.477	0.447	0.355	0.298	0.249	0.549	0.362	0.403	0.386	0.304	0.477	0.286	0.279	0.406
Indeno[1,2,3-*cd*]pyrene	2.458	2.455	2.482	2.459	2.584	2.477	2.504	2.410	2.148	2.104	1.749	2.672	2.201	2.133	2.239	2.004	2.412	1.936	1.940	2.413
Dibenzo[*a,h*]anthracene	0.048	0.043	0.047	0.050	0.044	0.051	0.052	0.046	0.076	0.039	0.039	0.162	0.061	0.075	0.092	0.074	0.111	0.065	0.061	0.030
Benzo[*ghi*]perylene	13.661	13.913	13.568	13.220	13.981	13.558	13.544	13.076	12.698	12.120	12.614	13.793	13.364	12.715	10.874	10.265	11.367	9.776	10.206	10.504
Anthanthrene	4.403	4.314	4.328	4.248	4.741	4.446	4.345	4.404	3.401	2.961	2.325	3.774	3.441	3.421	2.655	2.566	3.099	2.414	2.456	3.727
Coronene	11.327	11.319	10.756	10.695	11.561	11.407	11.158	11.094	11.317	9.549	8.880	12.197	11.529	9.851	10.893	10.169	10.787	9.430	9.999	12.140

Table S3. Overview of all individual measurement results for the PAH content of truck tires, whole tires (TW) in mg/kg (part 1)

Date	08.17		09.17		10.17		11.17		12.17		01.18		02.18		03.18		04.18	
**Sample number**	1.1 (A)	1.1 (B)	1.2 (A)	1.2 (B)	1.3 (A)	1.3 (B)	1.4	1.5	1.6 (A)	1.6 (B)	1.8 (A)	1.8 (B)	1.8 (C)	1.8 (D)	1.9 (A)	1.9 (B)	1.10 (A)	1.10 (B)
**PAH species**	*mg/kg*	*mg/kg*	*mg/kg*	*mg/kg*	*mg/kg*	*mg/kg*	*mg/kg*	*mg/kg*	*mg/kg*	*mg/kg*	*mg/kg*	*mg/kg*	*mg/kg*	*mg/kg*	*mg/kg*	*mg/kg*	*mg/kg*	*mg/kg*
Naphthalene	2.073	2.123	1.906	2.067	2.496	2.419	2.517	2.517	2.571	2.085	2.568	2.958	2.655	2.060	2.214	2.440	2.494	3.642
Acenaphthylene	2.168	2.188	1.480	1.818	2.296	2.276	2.245	2.160	2.410	2.137	2.688	2.692	2.544	2.347	2.248	2.509	2.364	2.559
Acenaphthene	0.208	0.206	0.117	0.126	0.155	0.161	0.162	0.210	0.182	0.170	0.236	0.463	0.196	0.317	0.251	0.245	0.284	0.300
Fluorene	0.377	0.383	0.274	0.307	0.317	0.323	0.328	0.439	0.381	0.354	0.374	0.716	0.360	0.546	0.435	0.425	0.528	0.512
Phenanthrene	5.851	5.941	4.042	4.900	5.263	5.017	5.352	6.286	5.795	5.476	5.878	5.650	5.545	5.247	5.321	5.608	5.262	5.579
Anthracene	0.492	0.560	0.291	0.350	0.351	0.344	0.385	0.464	0.438	0.444	0.456	0.461	0.397	0.403	0.378	0.400	0.400	0.462
Fluoranthene	9.665	9.550	7.821	8.766	9.298	9.066	9.401	9.619	9.586	9.003	10.172	9.589	9.731	9.241	9.338	9.638	9.314	9.612
Pyrene	35.390	34.484	30.103	33.494	34.960	34.849	34.960	35.796	35.774	33.669	36.103	34.166	35.267	33.513	33.494	34.478	33.765	34.235
Benzo[*b*]naphtho[2,1-*d*]thiophene	0.233	0.278	0.259	0.275	0.260	0.248	0.264	0.203	0.256	0.247	0.242	0.249	0.301	0.267	0.243	0.247	0.271	0.268
Benzo[*ghi*]fluoranthene	3.497	3.637	3.251	3.462	3.462	3.506	3.292	3.350	3.563	3.391	3.514	3.322	3.558	3.313	3.255	3.359	3.322	3.478
Benzo[*c*]phenanthrene	0.186	0.208	0.169	0.179	0.167	0.170	0.170	0.208	0.190	0.184	0.190	0.180	0.189	0.187	0.180	0.184	0.184	0.188
Benzo[*a*]anthracene	0.321	0.391	0.307	0.419	0.368	0.354	0.351	0.429	0.414	0.433	0.347	0.360	0.344	0.392	0.320	0.342	0.332	0.356
Cyclopenta[*cd*]pyrene	4.329	4.872	3.335	3.873	4.204	4.252	4.001	3.455	4.418	3.967	5.045	4.600	4.946	3.989	4.430	5.026	4.710	5.218
Triphenylene	0.341	0.377	0.362	0.411	0.415	0.377	0.402	0.306	0.368	0.379	0.380	0.397	0.431	0.449	0.393	0.410	0.386	0.345
Chrysene	0.502	0.583	0.484	0.617	0.588	0.534	0.568	0.574	0.604	0.617	0.515	0.543	0.541	0.600	0.505	0.530	0.517	0.508
Benzo[*b*]fluoranthene	0.492	0.636	0.473	0.521	0.554	0.503	0.536	0.490	0.552	0.550	0.578	0.555	0.567	0.590	0.537	0.552	0.559	0.561
Benzo[*k*]fluoranthene	0.148	0.196	0.125	0.162	0.146	0.153	0.163	0.120	0.153	0.152	0.180	0.160	0.169	0.161	0.149	0.166	0.185	0.190
Benzo[*j*]fluoranthene	0.157	0.211	0.145	0.157	0.167	0.155	0.158	0.142	0.173	0.170	0.194	0.186	0.185	0.189	0.170	0.178	0.195	0.193
Benzo[*e*]pyrene	2.011	2.362	1.995	2.142	2.192	2.080	2.116	1.948	2.140	2.163	2.301	2.277	2.378	2.387	2.181	2.278	2.237	2.213
Benzo[*a*]pyrene	1.617	1.906	1.536	1.703	1.768	1.697	1.649	1.724	1.725	1.755	1.871	1.858	1.810	1.882	1.756	1.848	1.768	1.763
Perylene	0.222	0.329	0.222	0.265	0.264	0.251	0.240	0.204	0.269	0.268	0.294	0.286	0.302	0.301	0.256	0.292	0.303	0.310
Indeno[1,2,3-*cd*]pyrene	1.860	2.204	1.787	1.767	1.899	1.827	1.736	1.642	1.957	1.897	2.317	2.058	2.150	1.993	2.006	2.142	2.035	2.044
Dibenzo[*a,h*]anthracene	0.016	0.027	0.021	0.027	0.022	0.023	0.025	0.019	0.029	0.032	0.029	0.023	0.024	0.029	0.020	0.027	0.023	0.026
Benzo[*ghi*]perylene	11.823	12.248	11.555	11.270	12.456	11.743	11.477	11.874	11.830	11.861	12.695	12.419	12.731	12.243	11.960	12.329	11.506	11.365
Anthanthrene	3.266	4.271	2.994	3.184	3.341	3.270	3.060	2.765	3.386	3.427	3.514	3.468	3.594	3.173	3.278	3.567	3.519	3.712
Coronene	10.666	10.478	10.399	9.994	11.247	10.625	10.180	10.318	10.819	10.823	9.424	9.453	9.795	9.121	9.274	9.604	8.813	8.811

Table S3. Overview of all individual measurement results for the PAH content of truck tires, whole tires (TW) in mg/kg (part 2)

**Date**	**05.18**		**06.18**		**07.18**		**08.18**	**09.18**	**10.18**	**11.18**	**12.18**	**01.19**	**02.19**	**03.19**	**04.19**	**05.19**	**06.19**	**07.19**
**Sample number**	1.11 (A)	1.11 (B)	1.12 (A)	1.12 (B)	1.13 (A)	1.13 (B)	1.14	1.15	1.16	1.17	1.18	1.19	1.20	1.21	1.22	1.23	1.24	1.25
**PAH species**	*mg/kg*	*mg/kg*	*mg/kg*	*mg/kg*	*mg/kg*	*mg/kg*	*mg/kg*	*mg/kg*	*mg/kg*	*mg/kg*	*mg/kg*	*mg/kg*	*mg/kg*	*mg/kg*	*mg/kg*	*mg/kg*	*mg/kg*	*mg/kg*
Naphthalene	2.763	2.622	2.121	2.207	2.606	2.547	2.025	2.272	2.253	2.207	2.341	2.270	1.805	1.917	2.166	2.152	2.711	2.210
Acenaphthylene	2.753	2.818	2.359	2.495	2.499	2.723	2.091	2.249	2.215	2.265	2.303	2.185	2.116	2.086	2.352	2.487	2.731	2.401
Acenaphthene	0.283	0.373	0.250	0.300	0.300	0.262	0.142	0.162	0.145	0.147	0.104	0.107	0.188	0.156	0.174	0.153	0.193	0.160
Fluorene	0.480	0.671	0.462	0.598	0.568	0.475	0.310	0.317	0.307	0.334	0.319	0.284	0.259	0.239	0.259	0.251	0.302	0.257
Phenanthrene	5.693	5.687	5.381	5.601	5.510	5.826	5.163	5.248	5.003	5.574	5.151	4.695	4.958	4.858	4.843	4.863	5.476	4.778
Anthracene	0.444	0.469	0.383	0.443	0.466	0.456	0.313	0.304	0.285	0.357	0.316	0.258	0.378	0.347	0.345	0.344	0.407	0.371
Fluoranthene	9.919	9.636	9.373	9.567	9.483	9.983	9.181	9.321	9.138	9.735	9.257	8.693	8.084	8.025	8.353	8.118	8.374	7.866
Pyrene	35.112	34.324	33.729	33.910	33.912	35.322	33.629	34.032	33.264	35.111	34.149	32.250	25.443	25.750	26.760	25.849	27.152	25.501
Benzo[*b*]naphtho[2,1-*d*]thiophene	0.244	0.243	0.240	0.249	0.231	0.254	0.187	0.165	0.170	0.172	0.164	0.187	0.167	0.174	0.191	0.173	0.254	0.170
Benzo[*ghi*]fluoranthene	3.463	3.225	3.249	3.351	3.310	3.414	2.928	2.915	2.947	3.133	2.928	2.813	2.694	2.808	2.870	2.976	2.961	2.870
Benzo[*c*]phenanthrene	0.195	0.185	0.184	0.205	0.186	0.185	0.151	0.148	0.151	0.158	0.145	0.145	0.145	0.146	0.144	0.150	0.171	0.162
Benzo[*a*]anthracene	0.443	0.448	0.348	0.409	0.406	0.413	0.303	0.281	0.292	0.348	0.356	0.326	0.317	0.312	0.283	0.283	0.343	0.308
Cyclopenta[*cd*]pyrene	5.171	4.686	4.224	4.442	4.712	5.092	3.614	3.838	3.867	4.465	3.727	3.681	3.051	3.025	3.176	3.690	3.615	3.138
Triphenylene	0.387	0.408	0.415	0.430	0.366	0.421	0.388	0.328	0.341	0.329	0.354	0.309	0.301	0.368	0.349	0.303	0.460	0.311
Chrysene	0.610	0.631	0.543	0.602	0.560	0.622	0.496	0.455	0.471	0.516	0.540	0.517	0.516	0.519	0.496	0.471	0.642	0.497
Benzo[*b*]fluoranthene	0.691	0.647	0.567	0.653	0.577	0.632	0.516	0.488	0.481	0.512	0.525	0.446	0.489	0.510	0.507	0.514	0.491	0.481
Benzo[*k*]fluoranthene	0.217	0.195	0.157	0.179	0.183	0.187	0.111	0.101	0.102	0.114	0.127	0.104	0.152	0.142	0.129	0.142	0.118	0.137
Benzo[*j*]fluoranthene	0.231	0.212	0.184	0.214	0.192	0.201	0.173	0.151	0.156	0.176	0.142	0.129	0.144	0.148	0.160	0.161	0.144	0.160
Benzo[*e*]pyrene	2.425	2.295	2.205	2.360	2.230	2.346	1.996	1.901	1.907	1.943	1.939	1.794	1.808	1.884	1.797	1.841	1.787	1.763
Benzo[*a*]pyrene	2.041	1.918	1.781	1.817	1.871	1.916	1.643	1.630	1.604	1.708	1.697	1.603	1.539	1.487	1.486	1.585	1.457	1.531
Perylene	0.363	0.305	0.250	0.290	0.278	0.298	0.214	0.193	0.202	0.217	0.219	0.186	0.235	0.223	0.212	0.243	0.231	0.206
Indeno[1,2,3-*cd*]pyrene	2.266	2.055	1.952	2.087	2.042	2.091	1.799	1.709	1.729	1.806	1.663	1.546	1.766	1.783	1.775	1.936	1.592	1.601
Dibenzo[*a,h*]anthracene	0.047	0.040	0.023	0.037	0.028	0.033	0.018	0.018	0.017	0.019	0.030	0.018	0.039	0.040	0.031	0.023	0.029	0.029
Benzo[*ghi*]perylene	12.456	11.873	12.317	12.242	12.065	12.359	12.084	12.039	11.767	12.033	11.781	11.131	9.176	9.546	9.521	10.299	9.419	9.294
Anthanthrene	3.835	3.294	3.048	3.182	3.379	3.464	2.483	2.438	2.573	2.887	2.419	2.273	1.823	1.815	1.772	2.428	1.888	1.790
Coronene	9.303	9.329	9.396	9.312	9.587	9.666	9.445	9.060	8.864	9.509	10.157	9.881	8.536	9.217	9.055	8.854	8.364	8.181

Table S4. Overview of all individual measurement results for the PAH content of truck tires, tread (TT) in mg/kg

**Date**	**07.18**	**08.18**	**09.18**	**10.18**	**11.18**	**12.18**	**01.19**	**02.19**	**03.19**	**04.19**	**05.19**	**06.19**	**07.19**	**08.19**		
**Sample number**	2.1	2.2	2.3	2.4	2.5	2.6	2.7	2.8	2.9	2.10	2.11	2.12	2.13	2.14		
**PAH species** **Material size**	< 0.4 mm													0.5–2.5mm		
Naphthalene	8.430	5.756	6.910	7.272	6.847	6.624	6.569	7.151	7.723	8.784	8.492	8.639	8.098	7.159	6.270	6.790
Acenaphthylene	5.950	4.454	5.523	5.584	5.103	4.579	4.873	5.266	5.619	6.096	5.872	5.803	4.830	5.055	4.204	4.801
Acenaphthene	0.168	0.146	0.211	0.140	0.196	0.143	0.147	0.265	0.246	0.356	0.264	0.229	0.183	0.106	0.109	0.099
Fluorene	0.327	0.278	0.342	0.290	0.318	0.289	0.259	0.587	0.557	0.628	0.473	0.453	0.388	0.252	0.238	0.257
Phenanthrene	8.460	7.389	8.368	7.349	7.820	7.337	6.954	8.675	8.015	9.004	8.520	8.634	8.692	7.396	6.644	6.832
Anthracene	0.642	0.506	0.610	0.486	0.567	0.540	0.482	0.606	0.578	0.653	0.617	0.645	0.669	0.444	0.387	0.391
Fluoranthene	15.981	13.053	14.157	13.494	12.575	13.093	12.258	15.164	15.000	15.919	15.283	15.633	15.980	11.717	10.786	11.643
Pyrene	47.817	37.907	40.491	39.482	37.418	37.928	35.906	44.787	45.063	47.044	45.275	46.635	47.042	32.740	31.227	32.735
Benzo[*b*]naphtho[2,1-*d*]thiophene	0.221	0.140	0.167	0.128	0.193	0.154	0.145	0.191	0.160	0.173	0.238	0.189	0.222	0.279	0.231	0.210
Benzo[*ghi*]fluoranthene	5.207	4.220	4.215	4.206	4.137	4.348	4.042	4.568	4.722	4.928	4.657	4.875	5.172	3.459	3.399	3.487
Benzo[*c*]phenanthrene	0.249	0.191	0.221	0.178	0.212	0.197	0.175	0.267	0.232	0.244	0.238	0.247	0.248	0.196	0.188	0.193
Benzo[*a*]anthracene	0.374	0.312	0.459	0.256	0.400	0.287	0.285	0.399	0.304	0.327	0.361	0.357	0.356	0.290	0.212	0.223
Cyclopenta[*cd*]pyrene	12.597	7.367	8.700	9.717	8.612	8.519	8.373	10.391	11.682	12.169	11.846	11.975	11.107	7.938	6.023	7.176
Triphenylene	0.276	0.271	0.373	0.206	0.353	0.241	0.262	0.299	0.219	0.240	0.306	0.235	0.234	0.333	0.284	0.274
Chrysene	0.535	0.477	0.710	0.421	0.600	0.446	0.462	0.600	0.439	0.456	0.537	0.484	0.452	0.661	0.508	0.465
Benzo[*b*]fluoranthene	0.973	0.754	0.809	0.689	0.772	0.797	0.673	0.843	0.816	0.888	0.912	0.909	1.079	0.716	0.589	0.585
Benzo[*k*]fluoranthene	0.283	0.191	0.215	0.177	0.214	0.209	0.167	0.241	0.240	0.247	0.284	0.289	0.320	0.190	0.162	0.138
Benzo[*j*]fluoranthene	0.300	0.210	0.207	0.195	0.246	0.215	0.221	0.280	0.243	0.293	0.314	0.323	0.386	0.202	0.186	0.165
Benzo[*e*]pyrene	2.841	2.494	2.489	2.311	2.693	2.732	2.295	2.852	2.807	3.095	3.006	3.065	3.589	2.350	2.086	2.007
Benzo[*a*]pyrene	2.866	2.341	2.466	2.317	2.355	2.488	2.106	2.772	2.771	3.131	2.954	2.934	3.375	2.098	1.664	1.776
Perylene	0.434	0.302	0.289	0.271	0.378	0.393	0.296	0.322	0.324	0.415	0.458	0.472	0.571	0.274	0.251	0.222
Indeno[1,2,3-*cd*]pyrene	3.429	2.806	2.691	2.738	3.090	3.319	2.657	2.892	3.054	3.277	3.326	3.462	4.216	1.851	1.571	1.529
Dibenzo[*a,h*]anthracene	0.025	0.024	0.033	0.014	0.035	0.022	0.018	0.018	0.013	0.013	0.018	0.023	0.015	<0,001	<0,001	<0,001
Benzo[*ghi*]perylene	15.512	13.839	13.191	13.166	12.884	13.939	11.975	16.491	16.863	17.420	15.666	16.314	19.272	11.610	9.942	10.449
Anthanthrene	5.577	3.231	3.056	3.564	4.058	4.406	3.657	4.311	4.625	5.377	5.410	5.605	6.262	1.992	1.574	1.601
Coronene	13.269	12.806	11.396	10.559	10.211	12.393	10.483	12.708	12.685	13.559	11.353	12.343	14.268	8.742	7.866	7.835
